# Anticipatory Mourning and Narrative Meaning-Making in the Younger Breast Cancer Experience: An Application of the Meaning of Loss Codebook

**DOI:** 10.3390/bs12040093

**Published:** 2022-03-28

**Authors:** Maria Luisa Martino, Daniela Lemmo, Ines Testoni, Erika Iacona, Laura Pizzolato, Maria Francesca Freda, Robert A. Neimeyer

**Affiliations:** 1 Department of Humanities, Federico II University, 80133 Naples, Italy; daniela.lemmo@unina.it (D.L.); fmfreda@unina.it (M.F.F.); 2 FISPPA Department, University of Padua, 35131 Padova, Italy; ines.testoni@unipd.it (I.T.); erika.iacona@unipd.it (E.I.); laura.pizzolato@unipd.it (L.P.); 3 Emili Sagol Creative Arts Therapies Research Center, University of Haifa, Haifa 3498838, Israel; 4 Department of Psychology, University of Memphis, Memphis, TN 38152, USA; neimeyer@portlandinstitute.org; 5 Portland Institute for Loss and Transition, Portland, OR 97219, USA

**Keywords:** narrative, meaning-making, anticipatory mourning, loss, breast cancer, women under 50

## Abstract

Breast cancer (BC) in women under 50 is a potentially traumatic experience that can upset a woman’s life during a crucial phase of her lifespan. Anticipatory mourning linked to the diagnosis of BC can produce a series of inevitable losses similar to those of the bereaved. Narration can be one tool to construct meaning, to grow through the experience, and reconfigure time perspectives during and after the illness. The aim of this study was to apply the Meaning of Loss Codebook (MLC) to the narrative context of young women with BC. An ad hoc narrative interview was administered to 17 women at four times during the first year of treatment. A thematic analysis was performed using the MLC, adopting a bottom-up and top-down methodology. The results highlight the MLC’s usefulness in capturing the experiences of the women, allowing for a greater appreciation of the nuances of the meanings embodied in their narratives. The thematic categories grounded in the MLC cover the whole experience of BC during the first year of treatment, attesting to the possibility of extending the use of the MLC to observe the longitudinal elaboration of the psychic experience of BC in addition to its established validity in the context of bereavement and loss.

## 1. Introduction

### 1.1. The Psychological IMPACT of the Breast Cancer Experience in Young Women

The diagnosis and treatment of breast cancer (BC) in patients under the age of 50 are critical and potentially traumatic experiences that can upset a woman’s life during a crucial phase of her lifespan and the achievement of her goals [1,2]. The number of people diagnosed with cancer increases every year, with BC being the most common form in women. The highest incidence is in the 34- to 49-year age group [1,2], with an 87% survival rate. Despite the increasing number of women with BC under the age of 50, the psychological literature on this specific subject, although greatly required by vulnerable and at-risk women, still appears to be extremely limited [1,2]. The diagnosis, type of surgery (mastectomy or quadrantectomy), and type of treatment (radiotherapy, chemotherapy, or hormone therapy) can engender physical and psychological issues regarding body image, fertility, early menopause, sexuality, and interactions with partners and children [3,4,5,6,7,8]. Studies link the risk of major traumatic outcomes to a more difficult psychological adaptation undermined by the fear of recurrence, a construct which has been particularly extensively investigated [9,10,11,12]. In contrast, the literature also highlights the opportunities for young women to experience personal growth during illness, transforming negative emotions into strengths, and modifying life priorities [13]. Another relevant aspect connects the psychological impact of the illness to the specific characteristics of cancer as a stressor: the intangible and internal nature of the threat, the uncertainty about the disease outcome, the unpredictable trajectories, and the chronological aspects [14], which present recurrent stressors across different phases of the medical process [15,16,17,18]. These characteristics generate an accumulated burden of adversity, which may significantly affect later psychological functioning [19].

### 1.2. The Anticipatory Mourning Condition in the Breast Cancer Experience

Despite medical advances in diagnosis and high rates of survival after treatment, still today any confirmation of BC arouses associations with the idea of death and/or the anguish of death in the affected person and in the family, making BC a life-threatening illness with internal representations [20]. Death is an inevitable phenomenon in any individual’s life, but individuals with a potentially fatal disease such as cancer demonstrate a greater anxiety about mortality as compared to people with other chronic illnesses. Patients experience anxiety during screening, when receiving a diagnosis, while undergoing treatment, and, subsequently, anticipating a recurrence of the disease. Therefore, the diagnosis of BC generates a psychological condition described as “anticipatory mourning”, studied by Elisabeth Kübler-Ross [21] and then by Therese Rando [22] and Testoni et al. [23]. Anticipatory mourning, as described by Rando [22], is a phenomenon encompassing seven generic operations (grief and mourning, coping, interaction, psychosocial reorganization, planning, balancing conflicting demands, and facilitating an appropriate death), which, within a context of adaptational pressures caused by experiences of loss and trauma, is stimulated in response to the awareness of a life-threatening illness in oneself or a significant other and the recognition of associated losses in the past, present, and future. Within the perspective of clinical psychology, anticipatory mourning can be expressed by depression, a preoccupation with the loss, and, in the case of family and close associates, an anticipation of the personal adjustments necessary to live without the dying person. Due to this anxiety, those affected also report related physical problems such as pain, insomnia, and physical discomfort and a poor quality of life. If this anxiety of death is not addressed during the treatment path, it reduces the life expectancy of the individual [24] and undermines strategies of coping with and adapting to the treatment, in particular for women, who tend to exhibit a greater level of despair, anger, somatization, depression, and death anxiety than men [25].

### 1.3. Narrative Meaning-Making and Loss: The Process of Reconstruction and Growth

Within a narrative, constructivist perspective, the experience of cancer generates a crisis affecting the basic elements that regulate the relationship between the internal and external worlds [26,27,28], interrupting the sense of continuity of one’s life story over time. The crisis affects sense-making processes that support the individual’s personal life story and continuity of life [26,29,30,31,32]. This experience imposes a narrative urgency on the mind, activating the need to synthesize new meanings and promoting the organization and connection of different elements of the experience [26]. Therefore, the device responds naturally to the human being’s fundamental need [33,34] to experience a sense of continuity and coherence by constructing stories in an intersubjective space and culture [17,18,35,36,37]. Narration is an elective tool to construct a meaning-making [38] of the BC experience and to reconfigure time perspectives [32] during and after the illness [39,40]. Narration aims to support adaptation, integrate the event, construct resources, promote well-being, and activate coping strategies [31,41,42]. These processes can be considered transformative [34] in their discursive tendency toward the search for a configuration that allows the patient to make sense, even if temporarily, of the experience of illness [26]. Importantly, the creation of meaning not only alleviates pain and distress, but also facilitates growth and well-being in the aftermath of loss, in accordance with a constructivist perspective, in which one’s sense of self is established through the stories one constructs about oneself and the sharing of these stories with others. Experiences of loss can challenge the validity of a person’s core beliefs and undermine the coherence of the narrative. Individuals can, therefore, resolve the incongruity by engaging in one of two general processes of meaning-making: either assimilating the experience of loss into their pre-loss beliefs and self-narratives or adapting to it by reorganizing their beliefs and self-narratives [38]. The literature has also highlighted how traumatic experiences, such as those related to illness, can produce post-traumatic growth (PTG) in the individual. PTG refers to a positive change in personality following events perceived as tragic [43]. A study by Walsh and colleagues highlighted the central role of PTG in the experience of prostate cancer survival. The same result was also found in children with an oncological diagnosis, confirming that growth following an oncological diagnosis is also present in young children [44]. Finally, a meta-analysis of 51 studies reported an assessment of the relationship between post-traumatic stress disorder (PTSD), post-traumatic stress symptoms (PTSS), and PTG in cancer patients and survivors. It was found that the relationship between PTSD/PTSS and PTG is moderately positive and robust. There is some evidence that the threat of advanced cancer is more closely associated with growth, but none to support the hypothesis that a longer time duration from the moment of the cancer diagnosis allows survivors the opportunity to positively reinterpret or find meaning in the traumatic aspects of the disease, resulting in a greater growth experience [45]. Considering BC as a pathology that produces a series of inevitable losses with experiences similar to those of the bereaved, Neimeyer and his colleagues [46] highlighted how attributing meaning to loss can result in an important growth process. He has developed an alternative model of bereavement arguing that the reconstruction of meaning in response to loss is the central process in bereavement [46,47]. He adopts as his starting point the view of bereavement as a process of reconstructing meaning, in line with the broader constructivist approach to psychotherapy [48] from which he derives the idea that human beings are meaning makers: weavers of narratives that give thematic meaning to the salient plot structure of their lives [49]. Through the innovative exploitation of culturally available belief systems, individuals construct permeable and provisional meaning structures that help them interpret experiences, such as bereavement or illness in this case, coordinate their relationships with others, and organize their actions toward personally meaningful goals [50]. The main objective of this research was to apply the Meaning of Loss Codebook (MLC) created by Gillies et al. [51] to longitudinal narratives of BC women through an integration of bottom-up and top-down perspectives of analysis, investigating the experiences of women diagnosed with cancer, the coping strategies they use to deal with the disease, the resources they perceive in their context, and the meanings they attribute to the disease and the losses they are experiencing. In addition, we aimed to investigate the growth experiences that these women felt they have acquired.

## 2. Study Design

### 2.1. Sample and Data Collection

The research was conducted at the National Cancer Institute Fondazione G. Pascale of Naples, the Italian national referral center for the treatment of neoplastic illnesses. The women who took part in this research were identified from medical reports and assessed for suitability in accordance with the following criteria: eligibility criteria were first access to hospital before the age of 50 and a diagnosis of infiltrating ductal BC; exclusion criteria were metastatic disease (stage IV), neoadjuvant therapy, and psychotherapeutic treatment in progress. In the first phase of the research, 50 women were recruited at the pre-hospitalization phase. During the study, the total number of women undergoing the four longitudinal phases was 17 (see Table 1), all below 50 years of age (M = 44.47; SD = 3.87). They were examined, first, during the pre-hospitalization phase (T1: 40–60 days before surgery); secondly, during the post-operative counseling phase (T2: 30–40 days after surgery) when they received the histological examination results; thirdly, during the adjuvant therapy phase (T3: 25–35 days after the start of the therapy, differentiated into chemotherapy, radiotherapy, and hormone therapy); and, finally, at the follow-up phase (T4: first check-up after about 8 months). The dropout of women from the first phase of recruitment was due to changes in the hospital structures, a worsening of the cancer condition, a desire on the part of the patients not to continue, and a lack of available time. The meetings took place by means of face-to-face interviews in an ad hoc room of the hospital. The data were collected during the year 2018.

### 2.2. Consent and Remuneration

The research was conducted within the framework of the STAR Programme, financially supported by UniNA and Compagnia di San Paolo. The research was co-constructed in collaboration with the hospital’s psychology service and breast unit and approved by the Pascale National Cancer Institute’s medical committee. The study respected the American Psychological Association’s Ethical Principles and Code of Conduct as well as the principles of the Declaration of Helsinki.

The hospital’s psychology service provided a location and facilities for the monitoring meetings and the treatment of the women who wanted to continue receiving psychotherapeutic support over time. The participants were informed about the study’s aims and procedures and were assured that their participation was voluntary and that their responses would remain anonymous. The women volunteered to participate by providing an informed consent in a written form, with the hospital approving the privacy policy.

### 2.3. Instrument

We constructed an original ad hoc narrative interview, named the Early Breast Cancer-Processing Trauma Interview (EBC-PTI), to explore young women’s narrative meaning-making processes within the BC experience in every phase of their therapeutic path. The same narrative interview was proposed to the women during different stages of their treatment, T1, T2, T3, and T4. Every medical phase, constituting a turning point in the medical treatment protocol, reflects a potential turning point in the meaning of a woman’s relationship with BC over time and the psychiatric challenges involved [36].

Phase I: Facing the unknown. The woman is still undergoing a diagnostic investigation of a suspicious nodule.

Phase II: Impact of the criticality of the disease. The woman learns about the severity of her pathology (receiving a histological examination), undergoes surgery for the malignant nodule, and decides the therapeutic path to be taken.

Phase III: Relationship with a changed body identity. The woman is faced with post-operative chemotherapy or radiotherapy treatments that affect her relationship with her body.

Phase IV: Construction of a new continuity. The woman returns to the daily routine of her life, integrating the maintenance phase, which will last for at least 5 years. During Phase IV, the woman finds herself recovering spaces of autonomy and gradually reduces her dependence on the medical institution, attending only for follow-up.

The narrative interview involved nine open questions, which started with an initial request to narrate the disease experience from the moment it appeared until the time of the interview. Each question was intended as a narrative prompt designed to open up a construction of sense at each stage of the ongoing experience.

The interview was constructed to activate different ways of describing the narrative discourse.

There is an alternation between questions that open up episodic narratives (e.g., *I would like you to choose three words/adjectives/idioms that come to mind when thinking about this phase of the experience. Is there a particular event/episode to which you relate when you say…? We are interested in knowing what happened, where you were, who else was with you, what you felt, what you thought at the time…*) and semantic narratives (e.g., *People sometimes imagine or have ideas about why they got sick… do you have any idea about it or do you imagine anything?*), thus activating the different functions involved in narrative thinking.

The questions were ordered to allow a gradual immersion in the critical experience, opening, in the final parts, a space for dialogue on resources and changes (e.g., *Could you tell me if there is someone or something that you have felt to be particularly helpful? How? Who do you find yourself talking about it with? About our meeting… Could you tell me if there is one thing in particular that you feel you can draw on from going through this phase?*).

The interview was conducted in an ad hoc room of the hospital. It had an average duration of approximately 45 min and was recorded and then transcribed verbatim. It was conducted by two female psychologists who are experts in clinical psychology and narrative methodology. Having interviewers of the same gender represented a key aspect in promoting the narrative of the women. The researchers were young women themselves, a fact which allowed them to construct an empathetic exchange relationship with the patients. In order to protect their identities, the names of women used in this text are pseudonyms.

## 3. Qualitative Analysis

The study followed a qualitative research design. The interviews were recorded and transcribed, and the resulting texts were subjected to a thematic analysis in order to identify similarities and differences between the narratives and to highlight relevant issues emerging from the dialogue [52]. In particular, six main steps were followed: a preparatory organization, a creation of categories or themes, a coding of data, a verification of any emerging understanding, a search for alternative explanations, and, finally, the writing of the summary report [52,53].

The analysis process followed two phases: First, the texts were analyzed using predefined categories derived from the MLC developed by Gillies et al. [51] (a top-down approach); this allowed, secondly, further unexpected themes to emerge (a bottom-up approach).

The researchers, in the first phase of the analysis, used the categories created by Gillies and colleagues [51], who had compiled the MLC through a thematic analysis of bereaved people’s narratives, addressing issues such as spirituality, personal growth, changing perspectives and values, and the meaning attached to loss. The authors of the MLC [51] aimed to identify a coding system applicable to oral and written accounts of the bereavement experience, allowing them to identify links between attempts at meaning-making by the bereaved, any meanings made, and the adaptation to loss. This coding system was implemented through the qualitative analysis of the responses of 162 adults who had lost a loved one. The open coding of the data allowed the identification of 30 categories concerning emotions, cognitions, relationships and behaviors, values, and beliefs related to grief. To pursue the aim of applying the MLC to longitudinal breast cancer narratives, *ex ante*, the authors excluded from the analysis the following categories that did not represent the context of this study: Decedent Preparation for Death, Affirmation of the Deceased, Release from Suffering, Missing the Deceased, Memories, Time Together, Survivor Identity, Lost Identity, Lost Innocence, and Identity as Bereaved Person.

Through the bottom-up analysis, the authors highlighted specific ways of narrative articulation in which the categories of the MLC linked to the experience of breast cancer and to the different phases of the first year of the disease experience. This approach allowed for the emergence of unexpected themes from the narratives, in particular, specific ways in which the narratives articulated the MLC thematic categories that that did not derive from the structured hypotheses of the existing literature [53,54].

In order to make the analysis as objective as possible, the researchers carried out the analysis of the texts simultaneously and independently. Once the coding procedure was completed, they compared the texts. They were required to reach the same conclusions, with differences of opinion being resolved through careful discussion until there was unanimous agreement. Finally, an agreement was reached with two additional supervisors.

## 4. Results

Below you will the main categories emerged by the analysis of the narrative interviews declined by the thematic categories covered by MLC.

### 4.1. Category Area: Positioning of Breast Cancer in the Women’s Life

Within this first category area, the following thematic categories were included from the MLC. The authors also highlighted meaning transformations during the different phases of the medical treatment (see Table 2).

- *Coping*, related to the various strategies implemented by the participants to cope with the cancer.
- *Moving On*, invoked by the participants who, instead of breaking down, choose to respond constructively to the losses resulting from the disease.
- *Acceptance*, linked to the acceptance of the cancer onset and treatment and the consequent serenity.
- *Emotionality*, which refers to the expression of unspecified emotions (but not of a depressive or negative nature) and the increased contact with them after the diagnosis.
- *Negative Affect*, which refers to all negative experiences related to sadness, anger, guilt, sense of emptiness, and unease.
- *Regret*, reflects expressions of regret about something done or left undone.

#### 4.1.1. Emotionality and Negative Affects Thematic Categories

In the first phase of BC, before the negative diagnosis, many women report that the BC period is characterized by a great precariousness, a sense of suspended time accompanied by helplessness. Ginevra said, “Until we know what size it is, what reality it is, I don’t know what to think… You think all these good and bad thoughts but in fact you don’t know, we don’t know yet, we are still in limbo” [PHASE I]. Zaira also used the word “limbo” to describe her situation: “It’s strange, I’m in a limbo. If all goes well then it must have been just a scare, I hope so. But inside of me I don’t feel it, I feel that maybe there really is something” [PHASE I]. The fear in the first phase is linked to any delay in waiting for the outcome of the diagnoses, and the fear is linked to the possible relapse at the end of medical treatment. For example, Ginevra said, crying: “You always have that fixed thought, it is always that, not knowing, the waiting, this phase, this waiting for these certain dates. It makes you nervous, it makes you feel tense, waiting for something you can’t control” [PHASE I]. The sense of precariousness and suspension follows two paths: the permanence of pervasive negative emotions and affects as in the case of Eleonora, who reported how, in all phases, she felt an enormous sadness that prevented her from carrying out her normal daily activities: “This experience is painful, just painful. I have changed, I am no longer the same as before, I do not do the things I did before even with my grandchildren. Enough, I don’t cook anymore, I don’t play with my grandchildren. This cancer has invaded everything” [PHASE IV]. Even after the recovery there remains the fear that the disease could return, as it did for Carolina: “My fear is: what if it returns? And what if you don’t get there in time?” [PHASE IV].

The transformation of negative affects and emotions toward the activation of internal resources to construct active coping strategies and to try moving on to counteract the initial passivity and fear caused by the onset of cancer affects some women, as we see in the following categories.

#### 4.1.2. Coping and Moving on Thematic Categories

Some women immediately felt the need to react to the BC. Marta said: “I’m afraid but I also have the courage to face the disease” [PHASE I]. Almost all the participants reported that they felt an initial fear that then gave way to the desire not to break down. They all said that they were committed to fighting the disease. Ginevra stated that “I found myself in a vortex but still reactive. I certainly didn’t get down about it, that’s a little, but sure” [PHASE III]. In particular, the women appeared to be centered on actions connected to the present time but with an internal future perspective to guide their battle. Lia [PHASE I] said “I have to fight, I want to take my life back in hand… I have to do it for myself and for my children”. Furthermore, women seemed engaged to find their active response strategies to the cancer, as Ludovica [PHASE IV] said, “Let’s see day by day, one step at a time and we’ll make it”.

#### 4.1.3. Acceptance Thematic Category

It is interesting to observe how, for some women, during the last phase of the therapeutic treatment for BC, it was also possible to achieve a sense of acceptance of the diagnosis. It was not a passive acceptance but rather was linked to the perception of being able to face the disease. Barbara said: “I can’t speak of resignation, but I feel a sense of acceptance of it. I am quite calm” [PHASE IV]. Carolina also hoped that everything would turn out for the best: “I’m happy because I’m happy. I’m also proud because I did it. And then there is also hope. I’m also hopeful of what’s to come, of course. There is also fear, that is always there” [PHASE IV].

#### 4.1.4. Regret Thematic Category

During the chemo- and radiotherapies (PHASES II and III), characterized by the losses of body identity, some participants also narrated the regret at not having undergone diagnostic tests earlier or at having underestimated the symptoms. The narration of regret was based on anger, anger directed toward themselves: “I think I was perhaps a bit negligent anyway, because initially I had this little peanut, but I turned to the primary care doctor and when I told him he said, oh well, the gland comes and goes. It is connected to the cycle anyway. I perhaps think that the fact was that I was not interested anyway, that is, I heard his opinion and did not investigate it, I could have avoided it. If action had been taken earlier it would not have become such a burdensome situation. I reproach myself for this, for, as we say, I trusted the opinion of a doctor. I didn’t go into this deeply at all.” [Miriam, PHASE II]. However, the participants also spoke of an anger not directed towards a specific person but aimed at the situation in general. For example, Carolina stated: “I have to do it again, the chemo, which I didn’t want to do. Now I’m angry” [PHASE II].

These attitudes were also influenced by previous experiences of family members’ illnesses, as highlighted by the bottom-up analysis. For example, Ginevra described a “mother with various cysts, grandmother with a mastectomy and then the removal of the breast and then the swelling and then the operation and then the amputation, and my aunt with her abdomen… so, in short, more or less… chemo, radio, post-operative and so on… You don’t know what the traumas will be that will accompany you from adolescence… even before, practically as a child and therefore, then… it’s all a film and you say “I’ve already seen this” and then… you worry about it.” Eleonora also reported how her experience of illness evoked the pain of the loss of her brother: “I felt a pain similar to this for the loss of my brother, yes… it is a pain… oh no, similar, because the loss of… I can’t compare the loss of a brother to this thing because in the meantime I’m still here talking about it and my brother is underground, but… like that, no” [PHASE II].

### 4.2. Category Area: Resources during the Treatment for BC

Within this second category area, the following thematic categories were included from the MLC. The authors also highlighted meaning transformations during different phases of the medical treatment (see Table 2).

- *Family Bonds*, which relate to changes in the family structure.
- *Valuing Relationships*, or the enhancement of relationships intended as resources to cope with the BC.
- *Spirituality*, linked to any mention of God, religion, spiritual faith.

#### 4.2.1. Family Bonds and Valuing Relationships Thematic Categories

The participants also answered questions related to the elements of their life that took on a supportive function during all stages of the disease. They also highlighted meaning transformations during different phases of the medical treatment. The support of the family plays a central role during all the phases of the BC treatment for most women. Sophie said: “My family has been close to me, my daughters and my husband, my father and my father’s second wife, let’s say she’s like a mother, in the sense that she’s always with me, she accompanies me in everything. When I found out now that I might have another tumor, the first person I called was my father” [PHASE I]. Most of the participants found support, especially within the family. Ginevra, answering the question about what helped her during her illness, replied “The whole family, they never left me” [PHASE IV]. Similarly, Ludovica replied: “My family has helped me in every way. My husband and my children. They have made me rest and stay calm” [PHASE IV]. All phases of the period of illness were also configured as an opportunity to enhance relationships outside the family system, with friends, relatives, and community. Artemisia talked about a new friendship with a woman who shared the same path of illness: “There was a person in the hospital who had a big problem, so much, so serious. She was a force, she was the one who helped me. She too had her problems but she helped me, she made me think positively” [PHASE IV]. Lara felt supported by her church group: “I find myself talking to the community I attend. They pray for me knowing that I have to have an operation. They are close to me with one little word” [PHASE II]. Barbara also felt welcomed by the community to which she belongs: “I have received support from the people, the shopkeepers, the people in my apartment block. Because at this time, being an architect, I had a job supervising a construction project in the building for the frame. The company found out about it, the neighbors in the apartment block found out about it. A total closeness from everyone. Solidarity and affection” [PHASE II]. Finally, the role of the team that monitored them was also recognized, as Ginevra stated: “I have never been alone, I have always been guided by my family, by the team” [PHASE II].

Some participants said that they had had to rely mainly on themselves. For example, Miriam: “I felt like I myself was my main helper. I strengthened myself, because I realized that in the end I couldn’t rely on people anyway, because others suffered too. I tried not to pour my problems onto others but to pour them onto myself” [PHASE IV].

#### 4.2.2. Spirituality Thematic Category

During the first phase, to cope with the high impact of the onset of the BC, three participants talked about the role of religion and prayer, which was fundamental to them in order to avoid becoming disheartened. For example, Lara said: “Approaching the Lord I have overcome many obstacles” [PHASE I]. Similarly, Lia confirmed the importance of faith in the healing process: “I understand faith as a support, believing in something that gives you the strength to then overcome some difficulty” [PHASE I]. In the same phase, one woman spoke of an anger also directed toward God, who had made her sick. This resulted in her losing her religious faith. Martha said: “I have withdrawn from the church. I no longer go to mass. I barely enter for the sign of the cross. It really upsets me. I tell the Lord, when you want it to end, let me know. I’ll be tough, but I’m also angry” [PHASE III].

### 4.3. Category Area: Post-Traumatic Growth

Within this third category area, the following thematic categories were included from the MLC. The authors also highlighted meaning transformations during different phases of the medical treatment (see Table 2).

Although the experience of BC was difficult for all the participants in that it caused a complete reorganization of their lives, most of them also highlighted the benefits deriving from the dramatic situation. The disease had, in fact, enabled them to realize a change of perspective and a redefinition of their priorities.

- *Valuing Life*, understood as the individual’s ability to give a new value to her life following the disease.
- *Living to the Fullest*, which refers to the desire of the participants to take advantage of the time left to them.
- *Impermanence*, linked to the fact that life is short and that everyone must die sooner or later.
- *Personal Growth*, which is related to a positive change realized at the level of character.
- *Lifestyle Changes*, or changes in daily routine.
- *Greater Perspective*, which is linked to the ability not to be disturbed by minor events.
- *Identity change*, refers to the presence of change but without any specification of the nature of its change.
- *Compassion*, which refers to the transformation due to the illness of the individual in term of becoming more altruistic, sensitive, empathetic, and willing or able to help others.

These thematic categories characterized mainly the last phases of the medical treatment.

#### 4.3.1. Valuing Life and Living to the Fullest Thematic Category

Numerous participants highlighted how the disease had allowed them to value life more profoundly. This process started in a few cases from the first phase of the BC treatment and, for most women, took place during the final phases of the treatment, once the risk to one’s life had been overcome, in an even more articulated narrative form. Lara, in the initial phase of her illness, during the period of investigation of her suspicious lump, said: “You know you could also die when you have a tumor, right? And then I think that life begins to be seen in a different way, every day is a more beautiful day, you give more value to things” [PHASE I]. During the final phases of the medical treatment, Artemisia spoke of a change in her life, saying: “Now I live life more calmly. It is as if all of a sudden you understand what the most important things are, the value of life, of loved ones, of children. You see more clearly the precious assets you own, which you always take for granted in your daily life. Instead, life can change from one moment to the next and you need to be aware of what you have” [PHASE III]. Since the onset of the disease had made clear the possibility of dying, the participants reported that they wanted to make the most of the time they had left. The exaltation of life had made them understand the importance of no longer putting off their commitments and of living day by day without focusing on futile things. Alice stated, [PHASE IV]: “I understood the value of life, of health. One day you have it and another you may not have it. You have to do everything you can and want to do without putting anything off”.

#### 4.3.2. Personal Growth, Lifestyle Changes, Greater Perspective, Identity Change, and Compassion

The participants themselves talked about personal growth and compassion, referring to the path of their illness at the end of the medical treatment. In particular, what happened to them made them more predisposed to help others and to discover new areas of self: “Maybe today I am more sensitive, I feel the pain of others more deeply and therefore I tend to feel more deeply the need to do good deeds, to concretely help others if they need help, to do good deeds towards those in need, because now I know what it means”[Artemisia, PHASE III]. Sophie also agreed that the disease increased her desire to be supportive of others: “All experiences, however negative, must then represent a point of reflection and growth. I would like to help others too. There are many people who suffer, children who suffer” [PHASE IV]. Other participants, on the other hand, said they have learned to think not only of others but also of themselves, beginning to love themselves. Barbara, for example, said: “I want to try to have a healthy selfishness, therefore also to say no to certain things” [PHASE IV]. Miriam agreed: “I understand that I have to be more selfish to be honest. Because I’m a little selfless and instead I have realized that I have to love myself first. No one can rightly love you more than yourself” [PHASE IV]. Love for oneself decreased the tendency to neglect oneself and to neglect one’s own needs, including physical ones, fueling the awareness that prevention is fundamental. Lia and Silvia, as soon as they discovered the presence of suspicious masses, wanted to emphasize how important it is to undergo regular checks: “From a medical point of view, certainly I understood the importance of self-assessments. It is necessary to check oneself, to prevent disease”.

#### 4.3.3. Impermanence Thematic Category

This thematic category was narrated by the women through a reference to and a framing of the disease and possible death as part of life and of a life that can sometimes be too short. Rosa [III PHASE] said, “Disease is part of life and can happen earlier or later to everyone… some first and some later”.

### 4.4. Category Area: Meaning-Making of the BC Experience

Within this fourth category area, the following thematic categories were included from the MLC. The authors also highlighted meaning transformations during the different phases of the medical treatment (see Table 2).

- *Meaning Made*, relating to having found a meaning to the disease and having understood the subjective cause of its onset.
- *No Meaning*, which refers to the attempt made by some of the participants to avoid seeking a deeper meaning behind the cancer.
- *Lack of Understanding*, which refers to the attempt to make sense or meaning but having not found it or having given up on the attempt to do so.

#### Meaning Made, No Meaning, and Lack of Understanding Thematic Category

Concerning the meanings attributed to the onset of the disease, different nuances of meanings were recorded during the phases of the medical treatment.

*Phases I and II*. Most of the participants talked about how the disease was linked to hereditary and familial factors. Women narrated a process of withdrawal and reflection on themselves. The meaning of the current experience was situated within themselves, allocated to their own body, genes, and story. Ginevra said, “[…] by descent, as a family inheritance, is the only explanation you can give”. Ilary reported, “I had a mother who had breast cancer 10 years ago”. In the first phases, there was present also the attempt to find a meaning connected to the demand “why to me?”. Lia said, “I don’t know why, I don’t imagine anything, I don’t blame anything or anyone”…“It’s normal… maybe I’ll understand in the future”. *Phase II*. In the second phase most women formulated generic or external meanings of the disease. On the one hand, we observed an anchoring in the shared/cultural/taken for granted dimensions of meaning to make the world appear more stable and understandable, considering the illness as something that is a part of life and is connected to the whole, without particular causes. On the other hand, the narrative tended to remove causality from oneself, attributing it to uncontrollable external factors, identifying specific causes in fate, God, and the environment. They believed that the disease struck them due to simple misfortune, as, for example, Silvia, who noted: “In reality I think it’s just bad luck, that is, it happened that it was me rather than someone else who had this thing… I don’t think that there is a particular reason why a person gets sick. It happens. Unfortunately, it can happen in the course of your life. This can happen rather than ending up under a tram or having any other misfortune and… it’s part of life”. Other women positioned their meaning in an external way by attributing it to living in the Terra dei Fuochi (an area in Campania, Italy, with widespread burning of toxic waste), which may have increased the risk of developing tumors. For example, Artemisia said: “Unfortunately we live in a world where there is the Terra dei Fuochi so you have to get sick sooner or later, for what you eat, for the air that is not good, so you get sick for this reason”. Similarly, Rosa stated: “I don’t know, perhaps due to the climate, the air. Keep in mind that I live in the Terra dei Fuochi where there are those smoke emissions that have clearly come out of the former factory… I have them at 300 m as the crow flies… and therefore… I don’t know… sometimes”.

*Phase III.* Chemotherapy and radiotherapy created losses around bodily identity for most of the women and reinforced causal attributions within themselves and their hereditary and genetic history. Ginevra and Alice said: “Breast Cancer is a genetic illness…it is hereditary”…“the onset of breast cancer is inside the family history”.

*Phase IV*. The aforementioned meanings emerged more clearly and concentrated on two aspects.

- Three participants believed that the disease was the result of a divine punishment or, in any case, the result of the Lord’s will, such as Martha, who said, “Sometimes I think it is a punishment from the Lord… for what I do not know”.
- One person attributed the cause of the disease to smoking or to ways they could have made different choices, thus ascribing the cause to themselves. For example, Zaira blamed “smoking, because I smoke, or use the pill”. Others believed that there was no meaning or cause linked to the disease but that the tumor simply represented the “disease of the century”, as Eleonora defined it.

## 5. Discussion

The MLC [51] has proved to be well suited to capture and convey the experiences of women with BC, since, through a differentiation of the content of their interviews, it allows for a greater appreciation of the nuances of their meaning, while enabling for a general categorization of their responses. The use of the MLC in the coding process revealed differences in the way women construct a process of ‘meaning-making’, an active construction of the sense given to external events to which the individual can then adapt. Moreover, the participants in the four different phases attributed different meanings to their illness. According to some scholars [51], every loss involves a continuous process of negotiation, assimilation, accommodation, and substitution of new meanings that modify the structure of the orientation system. The categorization by Gillies and colleagues [51] highlights how an experience of loss and a contact with death can lead people to value life more highly [55]. Among our participants, phrases similar to those found in the study just cited were very common, referring to the belief that life is precious and, as such, should not be taken for granted. Testoni et al. [56] also highlighted how mature reflection on the issue of finitude allows people to place a greater value on life and focus more acutely on the goals that they would like to accomplish before they die. Another category in this study where our findings were in agreement with the observations of Gillies et al. [51] concerned the desire to live to the fullest. The stimulus of approaching death makes clear the mortality of the human condition, encouraging patients to realize that they do not have an infinite amount of time at their disposal and that they need to take advantage of the life left to them. This finding also emerged from a study by Testoni and colleagues [57], who reported that the awareness that life is not eternal leads people to use their time more productively. The third category that Gillies and colleagues [51] also identified in their study relates to impermanence. The authors highlight how the contact with death produces an awareness that everyone dies, that life is short, and that sometimes life confronts us with events we cannot control, such as illness. However, reflection on these issues is usually censored by society, so much so that death and illness have become taboo subjects [58]. As indicated in the MLC, almost all the participants reported feeling empowered by the experience of illness. After an initial shock, they believed that they had the strength to cope with the disease. Most studies seem to confirm this result, with only a few cancer survivors displaying a severe maladjustment caused by the cancer, a result which seems to be true even when children are affected [44]. In fact, severe symptoms have been found in less than 20% of total research samples [59]. Walsh and colleagues [60] highlighted how adapting to a physical trauma such as illness, an internal offender, is different from adjusting to a trauma with an external offender. Physical trauma brings into play totally different coping strategies [61]. In the present study, participants spoke precisely of increased strength, maturity, and a change of priorities and responsibility, as in the study of bereaved persons by Gillies et al. [51]. The change in lifestyle, a category of the MLC most prevalent among the BC women, concerns taking better care of their health. Women with lower levels of education might be expected to suffer more frequent development of cancer, because of the fact that they are likely to have fewer economic opportunities for treatment and prevention and little knowledge of the prodromal aspects of the disease [62]. Changes were also found within family ties, linked to a greater predisposition to value relationships. All the participants highlighted how the disease made them feel even closer to their family and encouraged them to value their relationships more profoundly. In fact, they reported, in line with the study of Gillies and colleagues [51], that they valued greatly the social support they received from friends and community. The participants also reported similar experiences to those noted by Gillies et al. [51] in relation to the category of compassion, which, in their words, related to becoming more altruistic, sensitive, empathetic, and willing and able to help others after loss. Relative to the ability to cope with problems resulting from the illness, the MLC categories “coping”, “moving forward”, and “greater perspective” were frequently invoked in the interviews with participants. These categories refer to all the adaptive means of resolving difficulties, progress being made over time despite obstacles, and to the fact that participants report perceiving a greater lightness in their daily life, no longer placing importance on trivial matters as they may have done in the past. The resources identified by the participants often included a reference to spirituality, which in the study of Gillies et al. [51] included any mention of God, religion, or spiritual faith. Research generally has shown that religiosity correlates positively with the ability to respond to stress, a greater resilience, and subjective well-being [63,64], also in the context of oncology, where the literature has highlighted the impact of religiosity on well-being, life satisfaction, and stress [65,66]. Religion and spiritual life appear to be closely related to health, particularly in relation to feelings of resignation toward the illness and quality of life [67]. However, as in research on “complicated spiritual grief” [68], a minority of participants felt greater insecurity in relationship with God, wondering whether their disease represented some form of divine punishment, as in the case of one women’s narration. On the other hand, with regard to the emotional sphere, the participants, after accepting the disease, reported feeling calm and peaceful and that any “negative affect”, as it is described in the MLC, is characteristic only of the early stages of the disease, although the fear of cancer recurrence (FCR) persisted even in the later stages. The literature also highlights how the disease contributes to stress in the lives of the women survivors, linked not only to the fear that the tumor will return but also to the economic expenses related to treatment and the fear of suffering social and occupational discrimination. In addition, the surgery to be faced causes significant changes in a woman’s body, creating a sense of discomfort and difficulty in exposing themselves physically [69]. FCR is one of the elements that most concerned survivors but, paradoxically, it is also one of the anxieties in respect to which there is little psychological support. To some degree, FCR is functional because it motivates follow-up and health-promoting behaviors. However, excessive FCR can impair quality of life through psychological distress, functional disability, and maladaptive behaviors, including a hypervigilance for the manifestation of symptoms that may indicate recurrence [68]. The participant’s ability to react to an illness also seemed to be associated with the search for a deeper meaning underlying the illness. The creation of meaning plays a central role in healing [1,2,17,18,36,69], in particular, in relation to healing from loss, in this case not related to the literal mourning of the death of a close figure. In fact, lower levels of healing detected in the aforementioned study were associated with higher levels of negative experiences, related to the inability to find meaning in the loss or personal benefits from the loss, as shown in the study of Holland and colleagues [70] undertaken with mourners. It is, therefore, essential to integrate the meaning of the loss, to make it understandable and consistent with the narrative of one’s life, and to design a new sense of self [70]. This objective was, indeed, achieved by some of the participants in this study, who appeared to have gained a greater confidence in themselves and their abilities.

## 6. Conclusions

In this research study, the MLC proved to be relevant and effective in terms of analyzing the experiences of women with BC. Over the course of the various phases of the disease, patients undergo a series of significant changes, physically, socially, and psychologically, and often experience real grief. The MLC was shown to be well suited to capture the experiences of women with BC [51], as, through a fine-grained analysis of the content of their interviews, it allows for a greater appreciation of the nuances of meaning, which might escape other systems, while allowing for a general categorization. The thematic categories incorporated in the MLC cover the whole experience of breast cancer during the first year of treatment, attesting to the possibility of extending the use of the MLC to longitudinally observe the psychic elaboration of the experience of breast cancer in addition to the validity of its use within the context of bereavement. The clinical implications of our study are twofold: on the one hand, the MLC in the context of breast cancer experience can be used as an explorative, narrative tool of the diachronic level of meaning-making of the experience during the different phases of treatment to observe the changes in the construction of meaning over time; on the other hand, it can be considered as a preventive tool to observe the phases of rigidity in the process of constructing the meaning of the experience and being able to propose a psychological setting focused on specific thematic areas aimed at supporting the flexibility of meaning in the relationship with the experience.

Furthermore, the flexibility of the MLC can be a useful tool for a study of the transformation of meanings during medical treatments more generally.

## 7. Limitations

We have to highlight two main limits of this study. First of all, we have to clarify that the narrative in a deep interview was constructed for a clinical–qualitative purpose. It does not yet have metric parameters, but the authors will work on this aspect in future study.

Furthermore, the small group of women who participated in the research, although it was followed in a qualitative–longitudinal way, appears a limit to reaching a full generalizability of findings. It is the intention of the authors to implement the recruitment of women also in other oncological hospitals to be able to confirm the findings that emerged in this study and to deepen the transformations of the categories during different phases of treatment.

## Figures and Tables

**Table 1:** Sociodemographic characteristics of the breast cancer patients monitored during the four phases of the medical treatment. Pseudonyms are used to protect patient confidentiality.

Name	Employment	Marital Status	Number of Children
Ginevra	Housewife	Married	2
Artemisia	Housewife	Married	2
Clara	Housewife	Married	1
Sophie	Employee	Single	0
Marta	Housewife	Married	1
Carolina	Employee	Married	1
Eleonora	Housewife	Married	2
Lara	Self-employed	Married	2
Barbara	Self-employed	Married	1
Miriam	Housewife	Married	3
Silvia	Employee	Single	2
Ilary	Employee	Single	3
Ludovica	Housewife	Married	2
Rosa	Housewife	Single	0
Zaira	Employee	Married	2
Alice	Employee	Separated	1
Lia	Employee	Married	1

**Table 2:** Categories area and thematic categories of MLC.

Category Area	Category Area	Category Area	Category Area
Positioning of breast cancer in the women’s life	Resources during the breast cancer treatment	Post-traumatic growth	Meaning-Making of the breast cancer experience
The thematic categories of the Meaning of Loss Codebook Included			
Coping	Family bonds	Valuing life	Meaning made
Moving on	Valuing the relationship	Living to the fullest	No meaning
Acceptance	Spirituality	Impermanence	Lack of understanding
Emotionality		Personal growth	
Negative affect		Lifestyle changes	
Regret		Greater perspective	
		Change identity	
		Compassion	

## Data Availability

The data presented in this study are available on request from the corresponding author. The data are not publicly available due to the protection of the privacy of the women involved.
